# Low-dimensional physics of clay particle size distribution and layer ordering

**DOI:** 10.1038/s41598-022-11036-8

**Published:** 2022-05-02

**Authors:** Yifeng Wang, Michael Wang

**Affiliations:** 1 Sandia National Laboratories, P.O. Box 5800, Albuquerque, NM 87185-0779 USA; 2 Department of Polymer Science and Engineering, University of Massachusetts, Amherst, MA 01003 USA

**Keywords:** Geochemistry, Two-dimensional materials

## Abstract

Clays are known for their small particle sizes and complex layer stacking. We show here that the limited dimension of clay particles arises from the lack of long-range order in low-dimensional systems. Because of its weak interlayer interaction, a clay mineral can be treated as two separate low-dimensional systems: a 2D system for individual phyllosilicate layers and a quasi-1D system for layer stacking. The layer stacking or ordering in an interstratified clay can be described by a 1D Ising model while the limited extension of individual phyllosilicate layers can be related to a 2D Berezinskii–Kosterlitz–Thouless transition. This treatment allows for a systematic prediction of clay particle size distributions and layer stacking as controlled by the physical and chemical conditions for mineral growth and transformation. Clay minerals provide a useful model system for studying a transition from a 1D to 3D system in crystal growth and for a nanoscale structural manipulation of a general type of layered materials.

## Introduction

**Figure 1:**
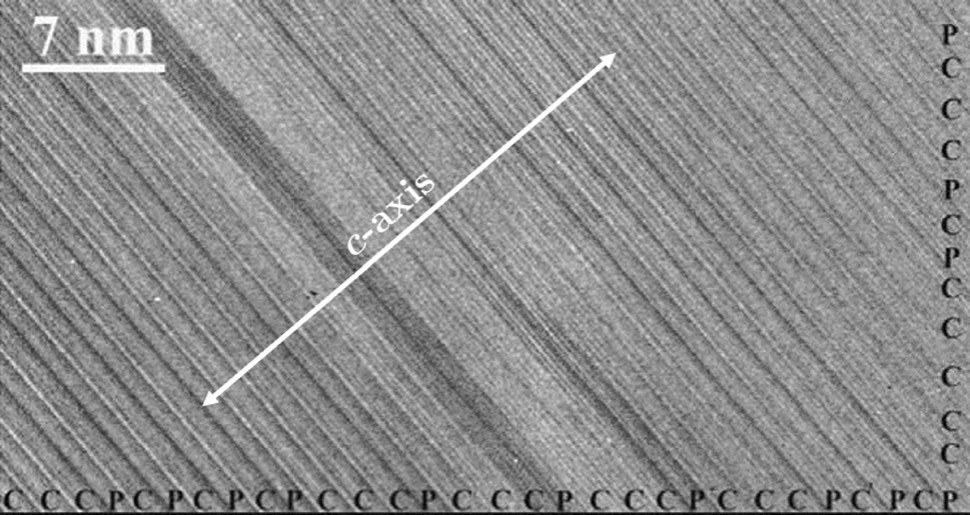
High resolution transmission electron microscope images of interstratified layers of chlorite (C) and pyrophyllite (P). Modified from Wang and Xu^7^.

Clays are ubiquitous in the Earth system, especially in sedimentary and weathering systems. Clays are layers of aluminosilicates (Fig. 1), in which one aluminum oxide octahedral sheet joins with one or two silica tetrahedral sheets to form what is called 1:1 (e.g. kaolinite) and 2:1 (e.g. smectite and illite) phyllosilicate layers. The thickness of a 2:1 layer is about 0.65 nm^1^. The Si and Al centers in the layers can partially be substituted by lower-valent metals, resulting in negative charges in the layers, which are then balanced by interlayer cations^2^. Clay are known for their small particle sizes and high density of defects^3^. The *a*-*b* dimension of clay crystallites ranges from a few nanometers to micrometers^4^, while the dimension along the *c*-direction ranges from $\sim 1$ to $\sim 100$ nm^3,5^. The dimension disparity between the two directions can be up to 200 times^1^. Based on the Periodic Bond Chains (PBCs) theory, Meunie^1^ suggested that the size and shape of a single clay platelet might depend on the amount of crystal defects along the three axes of symmetry [100], $[\bar{1}10]$, and $[\bar{1}\bar{1}0]$. Depending on cation ordering and occupancy in octahedral and tetrahedral sheets, crystal defects may tend to concentrate and thus poison crystal growth along one, two, or three PBCs, therefore limiting crystal dimensions in growth. The PBCs theory may provide a plausible explanation for the fibrous nature of some clay minerals such as sepiolite, but it fails to explain other key features of clay minerals such as the great dimensional disparity between illite and muscovite in spite of both minerals possessing a similar structure^6^.

**Figure 2:**
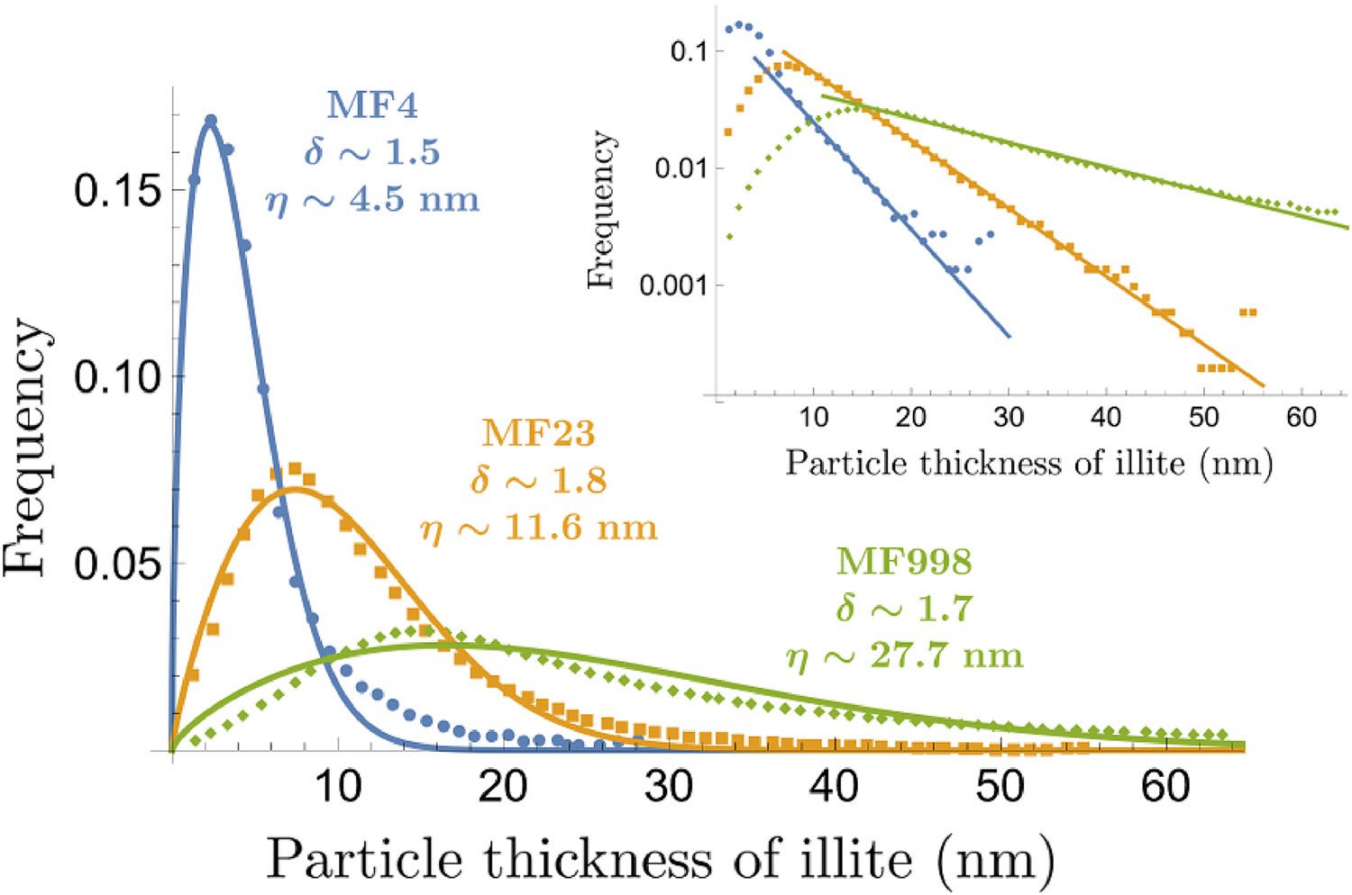
Clay particle thickness distributions for illites in shale from the Glarus Alps^5^. Metamorphic grade increases from sample MF4 (blue) to MF23 (orange) and to MF998 (green). Solid lines are fits to the Weibull distribution given by Eq. (22), where $\delta$ represents the effective dimensionality of the clay particles and $\eta$ represents the characteristic thickness. The inset shows a log-linear plot of the distributions, indicating nearly exponential tails in the clay particle size distribution for all the samples.

Under certain conditions, clays tend to form mixed layers with complex layer stacking patterns (see^7^ and refs. therein). For example, in the transformation of smectite to illite, the percentage of illite layers increases with temperature, geological time, and water/rock ratio, and accordingly the layer stacking mode shifts from R0 (random) to R1 (alternating), and then to longer-range order (R3)^4^. Based on a 1D Ising model, Zen^8^ attempted to provide a thermodynamic explanation for the formation of different layer stacking modes. By assuming that the interaction energy between layers depends only on the nearest neighbors, he showed that if the excess interaction energy between two unlike layers was large and positive, segregation into discrete crystals would result, and if the energy was large and negative, unlike layers would tend to alternate, forming a regular 1:1 mixed-layer crystal for equal proportions of the two layers. Intermediate energy values would result in irregular mixed layers, and a truly random layers would occur when the excess energy approaches to zero. In contrast, Wang and Xu^7^ suggested that the layer stacking would be a kinetic process and the sequence of layer stacking could be described by a one-dimensional logistic map, such that non-periodic interstratification emerges when the contacted solution becomes slightly supersaturated with respect to both structural components. The transition from one interstratification pattern to another reflects a change in the chemical environment during mineral crystallization. In all these models, the underlying assumption is that any ordered structure would extend to an infinite physical domain as commonly assumed for a crystalline system. With this assumption, one would hope that a mixed-layer clay can be modeled with a fixed composition and well-defined structure. However, as we show below, this assumption may no longer be appropriate for a clay system, in which a limited dimension becomes an inherent attribute of the material and the size of particles and the range of ordering are intimately related. Furthermore, no existing model can explain the observed layer thickness distribution along the *c*-direction of clay crystallites, which usually deviates from a lognormal distribution and highly skews towards small sizes (Fig. 2)^3,5^.

In this paper, we show that a clay mineral can be treated as two separate low-dimensional systems: a 2D system for the individual layers and a 1D system for the layer stacking. By formulating an appropriate statistical mechanical model for each system, we show that the dimension of clay particles is inevitably limited by the lack of long-range order in low-dimensional systems. This treatment will provide a new perspective on mineral phase definition and thermodynamic modeling of clay materials as well as the transition from a 1D or 2D system to a 3D system. Since layered minerals are a large set of materials with a wide range of applications in advanced technologies^9^, the work presented below will also provide an insight into the structural manipulation and synthesis of these materials.

## Results

### Clay as a low-dimensional system

The interlayer interaction in a clay mineral involves the electrostatic (electric double layer like), van der Waals, and hydration forces^10^, which are much weaker than the intralayer ionic/covalent bonding, leading to a significant anisotropy in mineral mechanical properties^11^. In an expansive clay such as smectite, multiple layers of water can exist in an interlayer. As the clay expands due to hydration, the interlayer interaction can become further weakened^12^, and the mineral can easily be exfoliated^13^. All these suggest that the growth of individual phyllosilicate layers can approximately be treated as a 2D system. Also, since the interlayer interaction is relatively uniform within the *a*-*b* plane, the layer stacking along the *c*-direction can be treated as a quasi-1D system.

It is well-known that low-dimensional systems (typically 1D or 2D) with short-range interactions generally do not exhibit a long-range order or phase transition. This behavior is often attributed to the Hohenberg-Mermin-Wagner (HMW) theorem^14,15^ for systems with continuous symmetries such as the XY model, to works by Landau and Lifshitz^16^ and Peierls and Born^17^ for systems with discrete symmetries such as the Ising model, and to van Hove^18^ for low-dimensional fluid-like systems. The typical explanation is that in low-dimensional systems, thermal fluctuations or other excitations have a strong tendency to disrupt any long-range order^19^. This result is quite universal and can be applied to a wide range of systems such as magnets, solids, superfluids, and membranes^20^. We postulate that this result can also apply to a clay system, that is, the limited dimension of a clay mineral is due to the lack of long-range order within its 2D layers and its 1D stacking of those layers.

With respect to individual phyllosilicate layers, much can be learned from the studies of engineered nanolayers. Nanolayers are solid layers with large in-plane dimensions but with nanometer thicknesses. Hong et al.^21^ studied the stability of ultrathin membranes of SrTiO$_3$ in epitaxial growth. Atomically controlled membranes were released after synthesis by dissolving the underlying epitaxial layer. Although all unreleased films were initially single-crystalline, the SrTiO$_3$ membrane lattice collapsed below a critical thickness. The authors showed that this crossover from power law to exponential decay of the crystalline order is analogous to the 2D Berezinkii-Kosterlitz-Thousless (BKT) transition. The BKT transition is a phase transition where the order in a 2D system of rotors such as the XY model is disrupted by the formation of unbound vortex and anti-vortex pairs^22^. The physics behind this behavior is quite universal and in the context of clay layers or 2D crystals, the lack of long-range order is due to the disruption of orientational order in a crystalline lattice. In this theory, one can define the correlation length of the crystalline lattice of a thin membrane. It is interesting to note that, similar to the process of a membrane released from a substrate, the expansion of clay interlayers through hydration could lead to a systematic reduction in clay particle size^23^.

If we assume that clay growth proceeds layer by layer, the BKT transition may take place within an individual phyllosilicate layer. For a weak interlayer interaction, a growing phyllosilicate layer would be constantly subjected to environmental fluctuations and any long-range structural order in the layers would be destroyed. Note that the thickness of a 2:1 phyllosilicate layer is about 0.65 nm^1^, thinner than the critical thickness for the BKT transition in an SrTiO$_3$ membrane^21^. As noted by Hong et al.^21^, the thermal fluctuations alone may be orders of magnitude lower than the energy required to break chemical bonds in a layer. However, the environmental fluctuations such as those in chemical potential and impurity concentration may be high enough to disrupt the lattice structure of a layer, leading to its limited extension.

### Layer stacking and the Ising model

We here develop a statistical mechanical model of an interstratified clay. Let us assume that an interstratified clay is formed by the stacking of two types of phyllosilicate layers, *A* and *B*. Note that in a more general context, one type of “layer” could not necessarily be a phyllosilicate layer and it can simply be a structural discontinuity or empty space. This can be useful if one wants to think of a system as a single type of clay that is fragmented. We further assume that the total energy of the system is determined by the interactions between nearest-neighbor layers. The Hamiltonian or energy *H* of this system can be expressed as

1 $$\begin{aligned} H=\frac{\epsilon _{AA}+\epsilon _{BB}-2\epsilon _{AB}}{4}\sum _{i=1}^{N}\sigma _i\sigma _{i+1}+\frac{\epsilon _{AA}-\epsilon _{BB}}{4}\sum _{i=1}^{N}(\sigma _i+\sigma _{i+1})+\frac{\epsilon _{AA}+\epsilon _{BB}+2\epsilon _{AB}}{4}N, \end{aligned}$$

where $\epsilon _{AA}$, $\epsilon _{BB}$, and $\epsilon _{AB}$ are the energies for the stacking of *AA*, *BB*, and *AB* layers, respectively; $\sigma _i$ is the type of layer *i* with $\sigma _i=1$ representing an *A* layer and $\sigma _i=-1$, a *B* layer; and *N* is the total number of layers in the system. Suppose that the mineral is in equilibrium with an aqueous solution of fixed chemical potentials $\mu _A$ and $\mu _B$ for layers *A* and *B* respectively. The partition function of the system can be written as

2 $$\begin{aligned} Z=\sum _{\varvec{\sigma }}e^{-\beta \left( H-\mu _AN_A-\mu _BN_B\right) }, \end{aligned}$$

where $\beta =1/kT$ is the inverse temperature, and $N_A$ and $N_B$ are the numbers of *A* and *B* layers respectively. The sum is over all combinations of layer types $\varvec{\sigma }=\{\sigma _i\}$. We can rewrite the numbers of each layer type as

3 $$\begin{aligned} N_A=\sum _{i=1}^{N}\frac{1+\sigma _i}{2},N_B=\sum _{i=1}^{N}\frac{1-\sigma _i}{2}. \end{aligned}$$

Note that this automatically enforces $N_A+N_B=N$. The partition function Eq. (2) can then be recast as

4 $$\begin{aligned} Z=\sum _{\varvec{\sigma }}e^{-\beta \left[ J_{\perp }\sum _{i}\sigma _i\sigma _{i+1}+\frac{K}{2}\sum _{i}(\sigma _i+\sigma _{i+1})+NH_0\right] }, \end{aligned}$$

where

5a $$\begin{aligned} J_{\perp }&=\frac{\epsilon _{AA}+\epsilon _{BB}-2\epsilon _{AB}}{2}, \end{aligned}$$

5b $$\begin{aligned} K&=\frac{\epsilon _{AA}-\epsilon _{BB}-\mu _A+\mu _B}{2}\end{aligned}$$

5c $$\begin{aligned} H_0&=\frac{\epsilon _{AA}+\epsilon _{BB}+2\epsilon _{AB}-\mu _A+\mu _B}{4}. \end{aligned}$$

Equation (4) resembles the standard 1D Ising model for material magnetization^24^ with an interaction energy $J_{\perp }$ and an external magnetic field *K*. Parameter $J_{\perp }$ controls whether like or unlike layers stack together, which depends on the interactions between two neighboring layers. *K* accounts for the difference between the two phyllosilicate components in the chemical affinity for clay layer precipitation from a contacted solution, which depends on solution chemistry. As an analogy, *K* represents the influence of an external chemical potential field. $H_0$ is simply a constant energy shift that will have no effect on the final results. We can evaluate the partition function using the transfer matrix method^25^. The major results are summarized as follows. The free energy of the interstratified clay is

6 $$\begin{aligned} F=-kT\ln Z=NH_0-kT\ln \left( \lambda _+^N+\lambda _-^N\right) , \end{aligned}$$

where $\lambda _{\pm }$ are the eigenvalues of the transfer matrix $$\left[ \begin{array}{ll} e^{\beta (J_{\perp }+K)} &{} e^{\beta J_{\perp }}\\ e^{\beta J_{\perp }} &{} e^{\beta (J_{\perp }-K)}\\ \end{array}\right]$$ given by

7 $$\begin{aligned} \lambda _{\pm }=e^{-\beta J_{\perp }}\cosh \beta K\pm \sqrt{e^{-2\beta J_{\perp }}\cosh ^2\beta K+2\sinh 2\beta J_{\perp }}. \end{aligned}$$

Note that $\lambda _+>\lambda _-$. In the thermodynamic limit ($N\rightarrow \infty$), we have $\lambda _+^N\gg \lambda _-^N$ and the free energy can be well-approximated by $F\simeq NH_0-NkT\ln \lambda _+$. The mean composition of layer *i* is

8 $$\begin{aligned} \langle \sigma _i\rangle =-\frac{1}{N}\frac{\partial F}{\partial K}=-\frac{e^{-2\beta J_{\perp }\sinh \beta K}}{\sqrt{1+e^{-4\beta J_{\perp }}\sinh ^2\beta K}}\equiv \cos 2\phi , \end{aligned}$$

where $\phi$ satisfies $\cot 2\phi =-e^{-2\beta J_{\perp }}\sinh \beta K$. A mean composition of $\langle \sigma _i\rangle =1$ means that all the layers are of type *A* while a mean composition of $\langle \sigma _i\rangle =-1$ means that all the layers are of type *B*. To quantify the structure or ordering of the layers, we compute the so-called two-point correlation function

9 $$\begin{aligned} \langle \sigma _i\sigma _j\rangle =\cos ^22\phi +\sin ^22\phi \left( \frac{\lambda _-}{\lambda _+}\right) ^{|j-i|}, \end{aligned}$$

and the correlation of fluctuations

10 $$\begin{aligned} \langle \delta \sigma _i\delta \sigma _j\rangle =\langle \sigma _i\sigma _j\rangle -\langle \sigma _i\rangle \langle \sigma _j\rangle =\sin ^22\phi \left( \frac{\lambda _-}{\lambda _+}\right) ^{|j-i|}, \end{aligned}$$

where $\delta \sigma _i=\sigma _i-\langle \sigma _i\rangle$ is a fluctuation of layer *i* from its mean composition. $\langle \sigma _i\sigma _j\rangle$ characterizes how correlated the type of layer *i* is with that of layer *j* while $\langle \delta \sigma _i\delta \sigma _j\rangle$ characterizes how correlated fluctuations about the mean type of layer *i* is with those of layer *j*.

**Figure 3:**
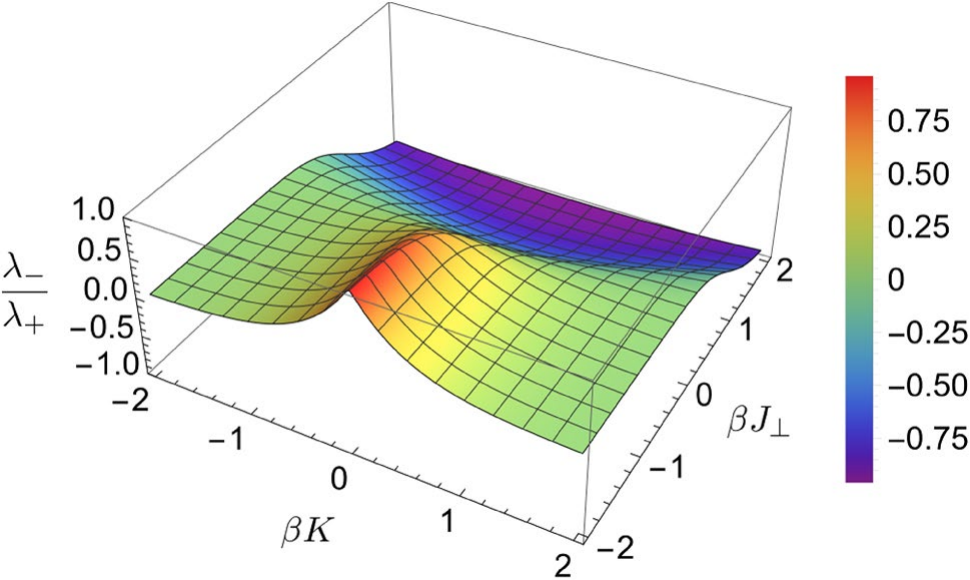
Ratio of eigenvalues [Eq. (7)] $\lambda _-/\lambda _+$, as a function of the interlayer interaction $J_{\perp }$ and external chemical field *K*.

Since $|\lambda _-/\lambda _+|\le 1$, the correlation functions given by Eq. (10) decays exponentially as the distance between two layers increases, which means that there is no long-range order in clay layer stacking. This suggests that we should abandom the existing attempt to model clay layer stacking as a long-range ordering process. The existing classification of R1 and R3 layer stacking modes should not be treated as long-range ordering patterns, but rather a local ordering phenonmenon.

It can be seen in Eq. (10) that the correlation of structural fluctuations is determined by parameters $J_{\perp }$ and *K*. As shown in Fig. 3, $J_{\perp }$ controls the sign of $\lambda _-/\lambda _+$ and therefore the layer stacking mode. A positive $J_{\perp }$ results in a negative ratio, leading to short-range alternating layer stacking, while a negative $J_{\perp }$ leads to short-range stacking of like layers. At $J_{\perp }=0$, we have random layer stacking since the ratio is zero and there are no correlations. A similar result was obtained by Zen^8^. $J_{\perp }$ also affects the magnitude of $\lambda _-/\lambda _+$ and therefore the correlation length in layer stacking; in particular, a larger $|J_{\perp }|$ generally enhances the length over which structural fluctuations are correlated.

As mentioned earlier, *K* represents the influence of the solution chemistry on clay layer stacking. Let us first consider the case when $K=0$, that is, there is no influence from the external chemical potential. In this case, the layer stacking is controlled only by structural fluctuations, and consequently the structural coherence length is equivalent to the correlation length of the fluctuations. From Eq. (10), the probability of a given coherence length of layer stacking exponentially decreases as the length increases. If we can consider this coherence length as the clay particle thickness, the clay particle size along the *c*-direction should follow an exponential distribution. Indeed, this exponential distribution of thickness or cluster sizes has been shown to be the case for Ising-like models with no external field^26^. For $K\ne 0$, increasing |*K*| causes one component to be enriched over the other in layer stacking, and as a result the particle size of the enriched component would increase. At the same time, as shown in Fig. 3, the ratio of $\lambda _-/\lambda _+$ approaches zero and so does the fluctuation correlation length. This means that a fluctuation of the type of one layer is not correlated with the fluctuations of the type of nearby layers. In this case, clay layer stacking is equivalent to a uniform random fragmentation process, in which the depleted component would randomly and uniformly insert into a sequence of layers of the enriched component. A uniform random fragmentation in a 1D system generates an exponential-like particle size distribution^27^. In all these cases, the particle size distribution along the *c*-distribution is thus predicted to follow an exponential or nearly exponential distribution. This is in qualitative agreement with actual measurements (Fig. 2)^3,5^ where it has been observed that the particle size distributions have exponential tails for large thicknesses. The exponential decay of correlation with length implies that the size of clay particles along the *c*-direction is finite. This is an inherent property of the one-dimensional nature of layer stacking, for which there is no long-range order.

For smaller thicknesses, as shown in Fig. 2, there is a considerable deviation from an exponential distribution (e.g. the peak in the distribution). However, it turns out that the distribution in this regime can still be described by a uniform random fragmentation process, but in a system with a dimension greater than one. In other words, while we may be able to treat the clay stacking as a one-dimensional process for large thicknesses along the *c*-direction, we may not be able to do so for smaller thicknesses. The distribution that arises from a uniform random fragmentation process in arbitrary dimensions is known as the Weibull distribution^27^. We will revisit this point in more detail in the “Discussion” section.

### Lateral dimension and the XY model

Now let us examine the stability of an individual phyllosilicate layer using an XY-like model. We define $\psi (\varvec{r})$ as the structural orientation field (i.e. the local orientational order of the crystalline lattice) in the tetrahedral and octahedral sheets. We here reproduce some key parts of the calculation of the fluctuations in the orientational order of a 2D lattice^20,22^. If the 2D lattice is ordered, the order parameter will be constant over the entire lattice or $\psi (\varvec{r})=\psi _0$. Due to environmental excitations, the lattice will deform and the orientational order will vary with position. Assuming that the gradients in $\psi (\varvec{r})$ are small, we can expand the Hamiltonian $H[\psi (\varvec{r})]$ to the second order in the gradient since $\varvec{\nabla }\psi (\varvec{r})\rightarrow -\varvec{\nabla }\psi (\varvec{r})$ should leave the energy unchanged, which gives

11 $$\begin{aligned} H[\psi (\varvec{r})]=\frac{J_{\parallel }}{2}\int d^2\varvec{r}[\varvec{\nabla }\psi (\varvec{r})]^2, \end{aligned}$$

where $J_{\parallel }$ is the interaction coefficient within a phyllosilicate layer. To make progress in computing the thermodynamic properties of this model, it is useful to express $\psi (\varvec{r})$ in Fourier space as

12 $$\begin{aligned} \psi (\varvec{r})=\int \frac{d^2\varvec{k}}{(2\pi )^2}\psi (\varvec{k})e^{i\varvec{k}\cdot \varvec{r}}, \end{aligned}$$

which gives us for the energy

13 $$\begin{aligned} H[\psi (\varvec{k})]=\frac{J_{\parallel }}{2}\int d^2\varvec{k}|\psi (\varvec{k})|^2k^2. \end{aligned}$$

The partition function of the system can then be expressed as an integral over all realizations of field $\psi (\varvec{k})$ given by

14 $$\begin{aligned} Z[\psi (\varvec{k})]=\int \mathcal {D}[\psi (\varvec{k})]e^{-\beta H[\psi (\varvec{k})]}=\int \mathcal {D}[\psi (\varvec{k})]e^{-\frac{\beta }{2}\int d^2\varvec{k}|\psi (\varvec{k})|^2\epsilon (\varvec{k})}, \end{aligned}$$

where $\epsilon (\varvec{k})=J_{\parallel }k^2$. The structural correlation function is defined as $c(|\varvec{r}-\varvec{r}'|)=\langle e^{i\psi (\varvec{r})}e^{-i\psi (\varvec{r}')}\rangle =e^{-\frac{1}{2}\left\langle \left[ \psi (\varvec{r})-\psi (\varvec{r}')\right] ^2\right\rangle }$, where the last step can be obtained by evaluating the average $\langle \cdot \rangle$ over realizations of field $\psi (\varvec{r})$ with the probability distribution $P[\psi (\varvec{r})]=Z^{-1}e^{-\beta H[\psi (\varvec{r})]}$. The last average $\left\langle \left[ \psi (\varvec{r})-\psi (\varvec{r}')\right] ^2\right\rangle$ can be computed as follows

15 $$\begin{aligned} \left\langle \left[ \psi (\varvec{r})-\psi (\varvec{r}')\right] ^2\right\rangle &=\int \frac{d^2\varvec{k}d^2\varvec{k}'}{(2\pi )^4}\left( e^{i\varvec{k}\cdot \varvec{r}}-e^{i\varvec{k}\cdot \varvec{r}'}\right) \left( e^{i\varvec{k}'\cdot \varvec{r}}-e^{i\varvec{k}'\cdot \varvec{r}'}\right) \langle \psi (\varvec{k})\psi (\varvec{k}')\rangle \\& =\int \frac{d^2\varvec{k}d^2\varvec{k}'}{(2\pi )^2}\left( e^{i\varvec{k}\cdot \varvec{r}}-e^{i\varvec{k}\cdot \varvec{r}'}\right) \left( e^{i\varvec{k}'\cdot \varvec{r}}-e^{i\varvec{k}'\cdot \varvec{r}'}\right) \frac{\delta (\varvec{k}+\varvec{k}')}{\beta \epsilon (\varvec{k})}\\& =\int \frac{d^2\varvec{k}}{2\pi ^2}\frac{1-\cos \left[ \left( \varvec{r}-\varvec{r}'\right) \cdot \varvec{k}\right] }{\beta \epsilon (\varvec{k})}\\& \approx \frac{1}{\pi \beta J_{\parallel }}\int _{\frac{1}{|\varvec{r}-\varvec{r}'|}}^{\frac{1}{a}}\frac{dk}{k}=\frac{1}{\pi \beta J'}\ln \frac{|\varvec{r}-\varvec{r}'|}{a}, \end{aligned}$$

where *a* is the lattice constant. Therefore, the structural correlation goes as

16 $$\begin{aligned} c(\varvec{r}-\varvec{r}')\approx \left( \frac{a}{|\varvec{r}-\varvec{r}'|}\right) ^{\frac{1}{2\pi \beta J_{\parallel }}}. 
\end{aligned}$$

This power-law dependence of the structural correlation on the distance between two points $\varvec{r}$ and $\varvec{r}'$ indicates that there is no true long-range order. In other words, over a large enough distance, the orientational order of the 2D lattice will be broken. This means that the structural coherence of a phyllosilicate layer is limited and so is the lateral dimension of the layer. For SrTiO$_3$ nanolayers (1.2–3.1 nm thick), after the layers were released from the growth substrate and freely suspended in water, the structural coherence length was estimated to be 4-40 nm^21^. Assuming that the bonding energy in a phyllosilicate layer is similar to that in an SrTiO$_3$ nanolayer, and considering that this energy can be further increased by the interactions between clay layers (see Section “Correlation between layer extension and clay composition”), we estimate that the structural coherence length of a clay layer could range from nanometers to micrometers, consistent with observations^4^.

The intralayer interaction can also be anisotropic depending on the lattice structure of a phyllosilicate layer. For example, in sepiolite, the chemical bonding in one direction is stronger than that in another. This would result in the structural coherence length to be longer along one direction, leading to the fibrous nature of the mineral. Indeed, we can note from Eq. (16) that the distance $\delta r^*=|\varvec{r}-\varvec{r}'|^*$ at which the structural correlation decays to a threshold value $c^*$ satisfies $\ln \frac{\delta r^*}{a}\approx 2\pi \beta J_{\parallel }\ln \frac{1}{c^*}\sim \beta J_{\parallel }$, which suggests that a small change in $J_{\parallel }$ can induce a large change in structural coherence and hence a strong structural anisotropy. In addition, Eq. (16) suggests that the area of clay platelets should follow a power-law distribution, which yet needs to be confirmed experimentally.

### Compositional variation of an interstratified clay

One challenge in modeling a mixed-layer clay is that such a mineral does not have a fixed stoichiometry in terms of chemical composition^28^, that is, the percentage makeup of the types of layers can vary from sample to sample. A common approach is to choose the appropriate layer types with fixed percentages and then use a solid solution model for layer mixing^29–33^, which is mostly empirical with many model parameters to be constrained. The benefit of our model is that it does not require any additional assumptions about the mixing and contains fewer parameters that can be directly related to the physics of the system. From Eq. (8), we can easily calculate the average molar fraction of component *A*, $X_A$, in an interstratified clay in equilibrium with a porewater as

17 $$\begin{aligned} X_A=\frac{1+\langle \sigma _i\rangle }{2}=\frac{1}{2}-\frac{e^{-2\beta J_{\perp }}\sinh \beta K}{2\sqrt{1+e^{-4\beta J_{\perp }}\sinh ^2\beta K}}. \end{aligned}$$

**Figure 4:**
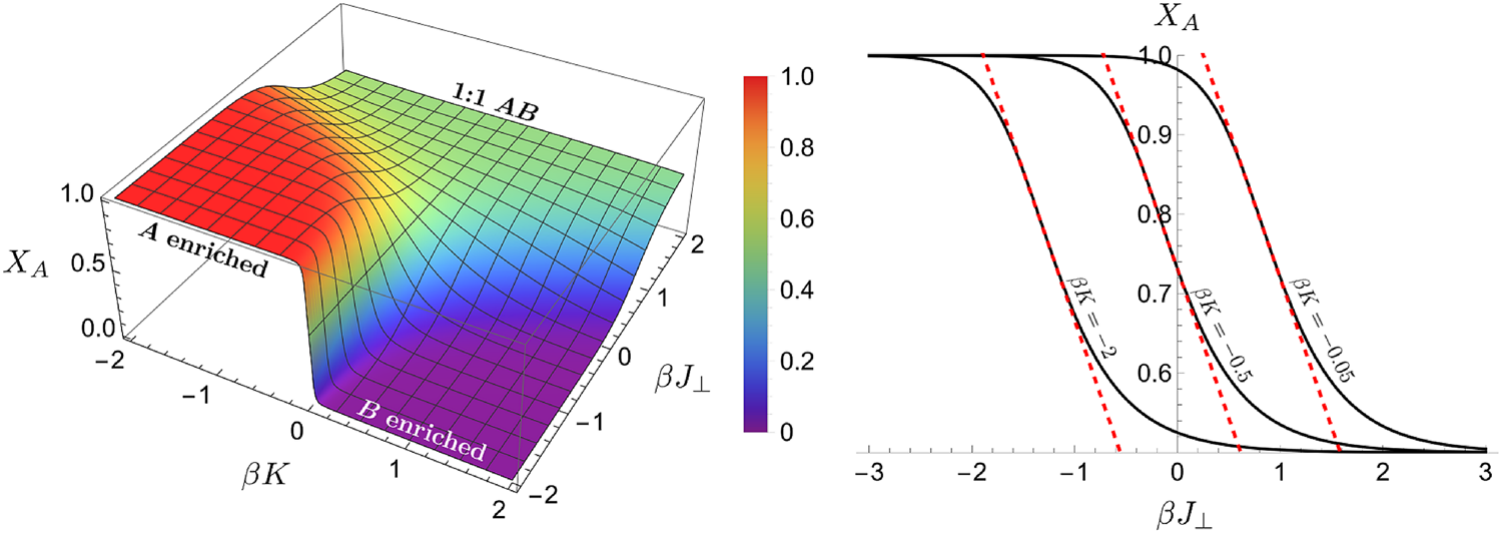
Molar fraction of layers of type *A* in a mixed-layer clay predicted as functions of parameters $J_{\perp }$ and *K*, which control the manner of stacking and mean composition, respectively.

This is plotted in Fig. 4. The composition of a mixed layer clay is thus determined by just two parameters: the interlayer interaction $J_{\perp }$ and the external chemical field *K*. Note that we can rewrite *K* as $K=K_{\epsilon }+K_{\mu }$, where $K_{\epsilon }=(\epsilon _{AA}-\epsilon _{BB})/2$ and $K_{\mu }=(\mu _B-\mu _A)/2$. By varying the solution chemistry (i.e. the chemical potentials $\mu _A$ and $\mu _B$) and measuring the layer composition [(Eq. (17)] and correlations [(Eqs. (9), (10)], one can easily determine the parameters $J_{\perp }$ and $K_{\epsilon }$. Once these two parameters are determined, the composition of the mineral can then be predicted for a whole range of solution chemistries. In this approach, we do not assume any long-range ordering in the clay minerals; instead, we treat a local clay particle aggregate as a single thermodynamic ensemble with short-range interactions and ordering. Such an approach may significantly simplify the way mixed-layer clays are modeled in water-rock interactions and allow for an easy prediction of various thermodynamic properties such as composition, Gibbs’ free energy, and mineral structure.

### Dimension disparity

As mentioned earlier, the *a*-*b* dimension of clay crystallites ranges from a few nanometers to micrometers^4^, while the *c* dimension ranges from $\sim 1$ to $\sim 100$ nm^3,5^. This dimension disparity between the two directions can be up to 200 times^1^. This may be attributed to the way how the structural correlation decays along the two directions. As indicated in Eqs. (10) and (16), the two-point structural correlation decays exponentially along the *c* direction while only follows a power law along an *a*-*b* direction. Since the former decays much faster than the latter, the dimension of clay crystallites along the *a*-*b* direction would be larger than that along the *c* direction.

### Correlation between layer extension and clay composition

**Figure 5:**
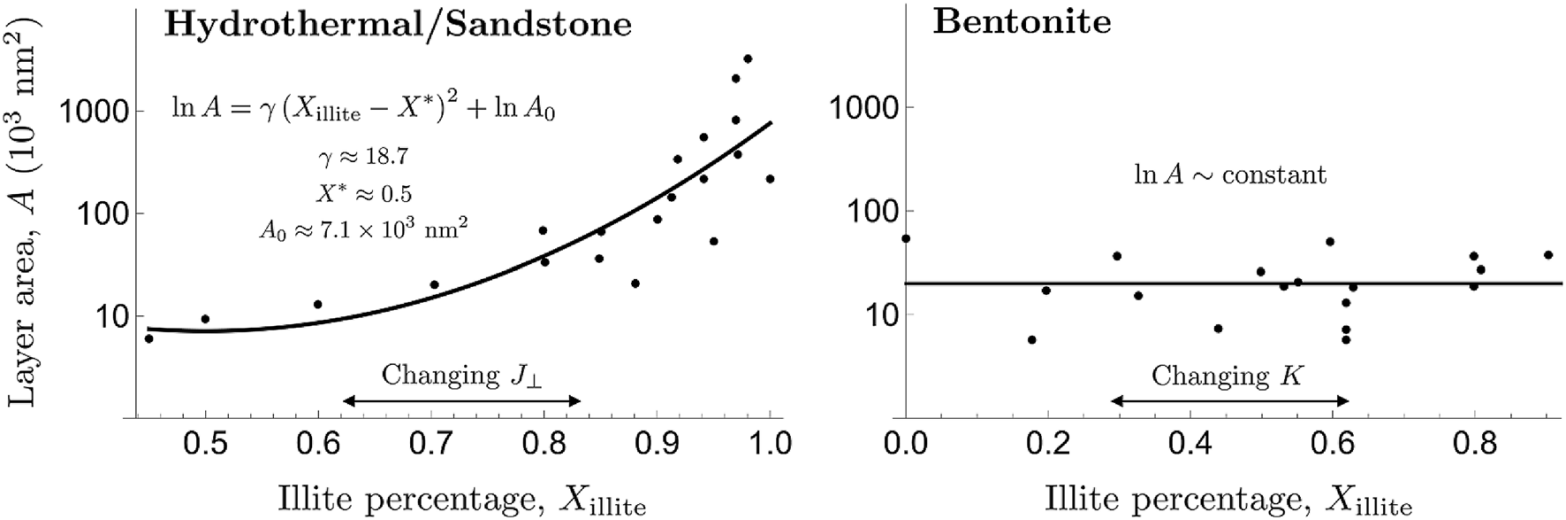
Layer areas in illite-smectite mixed layers from two different geological environments as a function of the percentage of illite. The solid lines are fits based on Eqs. (20) and (21). Data are taken from^4^.

Our model provides a reasonable explanation for the observed correlation between the lateral extension of clay platelets and the composition in mixed-layer samples^4^. As shown in Fig. 5, the area of illite layers in illite/smectite mixed layers strongly correlates with the percentage of illite in the samples from a hydrothermal/sandstone system. However, there is no such correlation at all in the samples from bentonite. To explain this difference, let us choose illite as component *A*. We argue that there should be some coupling between the interlayer interaction $J_{\perp }$ and the intralayer interaction $J_{\parallel }$. For example, the interaction with neighboring layers would reduce the freedom for layer structural fluctuations, which equivalently increases the interaction within the layers. Writing $J_{\parallel }=f(J_{\perp })$ and expanding about $J_{\perp }=0$, we have

18 $$\begin{aligned} J_{\parallel }=J_{\parallel ,0}+f'(0)J_{\perp }+\frac{1}{2}f''(0)J_{\perp }^2+\cdots , \end{aligned}$$

where $J_{\parallel ,0}$ is the interaction within a clay layer when there is a very weak interlayer interaction. As discussed earlier, the sign of $J_{\perp }$ determines whether like or unlike layers stack together. The influence of a neighboring layer on the intralayer interaction $J_{\parallel }$ of a given layer is expected to be independent of the type of the neighboring layer as long as the strength of interlayer interaction $|J_{\perp }|$ is the same. Therefore, we expect $J_{\parallel }$ to be an even function of $J_{\perp }$ and so $J_{\parallel }\simeq J_{\parallel ,0}+\frac{1}{2}f''(0)J_{\perp }^2$. As shown in Fig. 4, we can approximate $X_{\text {illite}}$ as a linear function of $J_{\perp }$ for a fixed *K* or

19 $$\begin{aligned} X_{\text {illite}}\simeq \frac{3}{4}-\frac{3}{8}\beta \left( J_{\perp }-J_{\perp }^*\right) , \end{aligned}$$

where $J_{\perp }^*=\frac{1}{2\beta }\ln \sinh \beta K$. Furthermore, by choosing the threshold value $c^*$ for the structural correlation given by Eq. (16), we can define the characteristic correlation length of a clay platelet $\delta r^*$ and area $A\sim {\delta r^*}^2$. From Eq. (16), we have that $\ln \delta r^*\sim \beta J_{\parallel }$, and thus $\ln A\sim \ln \delta r^*\sim J_{\parallel }$. Using the quadratic relation between $J_{\parallel }$ and $J_{\perp }$, we find

20 $$\begin{aligned} \ln A=\gamma J_{\perp }^2+\ln A_0, \end{aligned}$$

where $\gamma$ and $A_0$ are constants. The key result is that the area of the clay platelets is dependent on the interlayer interaction $J_{\perp }$ and not on the external chemical field *K*. If *K* is held fixed, we can invert Eq. (19) to find an approximate linear relation between $J_{\perp }$ and $X_{\text {illite}}$, which gives

21 $$\begin{aligned} \left. \ln A\right| _{K\text {fixed}}=\gamma '\left( X_{\text {illite}}-X^*\right) ^2+\ln A_0. \end{aligned}$$

Therefore, if the interlayer interaction $J_{\perp }$ is varied with the external chemical field *K* held fixed, there should be a strong correlation between the area *A* of illite layers and the mineralogical composition $X_{\text {illite}}$ of the clay. On the other hand, if the external chemical field *K* is varied with the interlayer interaction $J_{\perp }$ held fixed, the area of illite layers should be independent of the mineralogical composition. This is exactly what is observed in Fig. 5.

Changes in the interlayer interaction $J_{\perp }$ are mainly driven by variations of temperature and pressure^12^. Increasing the temperature would reduce the number of water layers in the clay interlayer, the basal spacing of the clay, and therefore the layer interaction energy of the clay^10,12,34^. Such environments are often observed in hydrothermal or sandstone systems and, as shown in Fig. 5, there is indeed a strong correlation between the layer area and the mineralogical composition. On the other hand, in low temperature environments such as surface weathering systems, the formation of clay is mainly driven by the chemical affinity of a contacted solution or changes in *K* while $J_{\perp }$ remains unchanged^35^. Bentonite is such a system, which, as shown in Fig. 5, exhibits almost no correlation between the layer area and the mineralogical composition. Thus, through simple scaling and symmetry arguments, we obtain a reasonable explanation for the correlations between the layer area and the mineralogical composition observed in various clay systems. In the more general context of 2D crystalline systems, the observed correlations between the interlayer interaction and the area of the layers may provide useful insight into the formation of thin materials with large lateral extensions.

## Discussion

**Figure 6:**
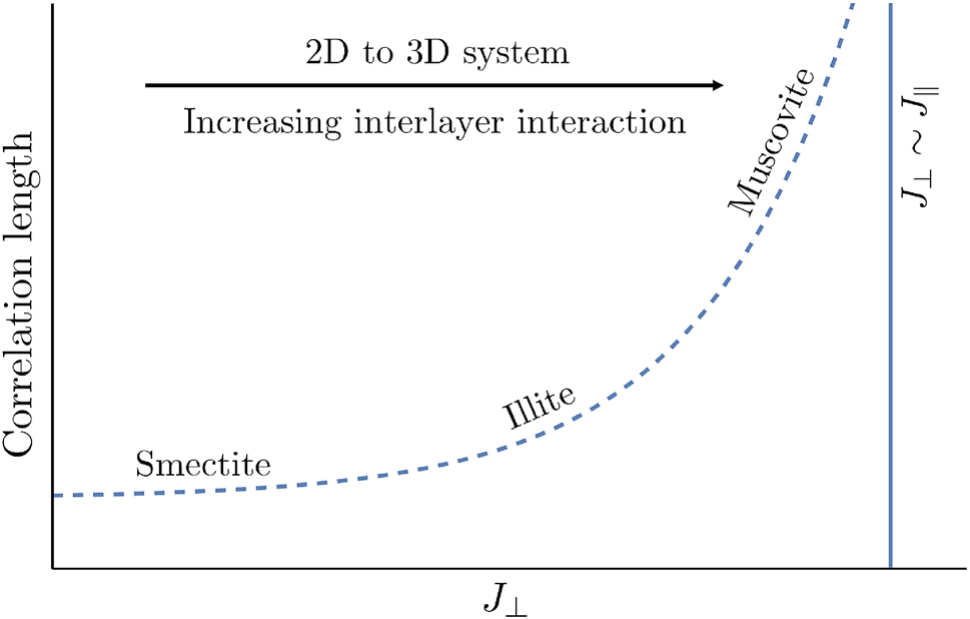
Schematic of the structural transition from a 2D system to a 3D system. As the interlayer interaction $J_{\perp }$ increases, the structural correlation length grows and at a certain point the clay system begins to behave as a 3D system instead of a 1D stack of 2D layers. Muscovite has stronger interlayer interactions than smectite or illite and is often observed to have much larger crystal sizes.

The effect of structural fluctuations in different dimensions has been illustrated in^36^. In a 1D lattice of particles with short range interactions, the relative fluctuations between the ends of a chain of *N* particles grows as $\sqrt{N}$ since fluctuations add up independently. This means that there cannot be any periodic structure over large distances in 1D at finite temperatures. In a 2D lattice, the fluctuations grow logarithmically with distance [e.g. Eq. (15)] while in a 3D lattice, they are finite over any distance. Therefore, for dimensions less than three, structural order cannot persist over large distances. This change in structural order as one transitions from a 2D to a 3D system should generally be observable in layered materials. At one end of the spectrum are clays, which have relatively weak interlayer interactions. As a result, each individual phyllosilicate layer can be treated as a 2D system and the lateral extension of the layer is then limited by the lack of long-range order. However, as the interlayer interaction increases, such as in muscovite^10,34^, a layered mineral may approach a 3D crystal system with a long-range order, resulting in the formation of large crystals. As formulated in Section “Correlation between layer extension and clay composition”, the intralayer interaction $J_{\parallel }$ should increase quadratically with the interlayer interaction $J_{\perp }$. Consequently, the correlation length should increase with the interlayer interaction. This is schematically illustrated in Fig. 6. This concept provides a plausible explanation for the observed great size disparity between illite and muscovite, both with a similar mineral structure, which cannot be explained based only on a mineral structure argument. Two major factors control the interlayer interaction of clay minerals: the layer charge and the temperature. The interlayer interaction is expected to increase with increasing the layer charge. The layer charge per cell unit of O$_{20}$(OH)$_4$ increases from smectite (0.5–1.2) to illite (1.4–2.0) and ultimately to muscovite (2.0)^37^, and so does the interlayer interaction. Furthermore, muscovite tends to occur in a high temperature environment. An elevated temperature would reduce the basal spacing of a clay mineral (i.e. the number of water layers)^38^ and the interlayer hydration through reducing the water dielectric constant^39^. All these effects combined would result in a strong interlayer interaction for muscovite. Given a strong (more than exponential!) dependence of the lateral extension of a clay layer on the interlayer interaction as predicted by Equation (20), it is reasonable to expect that the lateral extension of muscovite would be much larger than that of illite. The trend illustrated in Fig. 6 is consistent with the observed transition from smectite to illite and ultimately to muscovite in the prograde transition of mudstone to slate^3^. It is interesting to note that relatively large smectite crystals have been synthesized at high pressure and temperature^40^.

Up to this point, we have treated clay layer stacking as a 1D process. We have shown that the correlation of layer fluctuations along the *c*-direction for an enriched mineral [Eq. (10)] implies a uniform random fragmentation process in 1D and that the resulting particle size distribution should be exponential. Indeed, the distributions have exponential tails for large particle sizes (Fig. 2, inset). As noted, however, there is a considerable deviation from an exponential distribution for smaller particle sizes. One possible explanation for this deviation is that for smaller particle sizes or stacks of layers, a clay system may no longer be treated as a one-dimensional stack of layers but rather somewhere between one and two dimensions (a single layer is of course two-dimensional). Tenchov and Yanev^27^ generalized the 1D fragmentation result to higher dimensional systems. They showed that the particle size distribution generated from a uniform random fragmentation process in arbitrary dimensions is given by the Weibull distribution

22 $$\begin{aligned} P(d)=\frac{\delta }{\eta }\left( \frac{d}{\eta }\right) ^{\delta -1}e^{-\left( \frac{d}{\eta }\right) ^{\delta }}, \end{aligned}$$

where $\eta$ is the characteristic particle size (or the thickness of a structurally coherent clay crystallite along the *c*-direction) and $\delta$ is a constant characterizing the dimensionality of the fragmentation process. For $\delta =1$, Eq. (22) reduces to an exponential distribution. For smaller particle sizes, we expect the dimensionality of the fragmentation process to have an effective dimensionality $1\le \delta \le 2$. For $\delta >1$, the distribution becomes peaked towards smaller particle sizes, which is exactly what is observed in measurements of clay thickness distributions shown in Fig. 2. Fitting the distributions gives us dimensionalities $\delta$ ranging from 1.5 to 1.8 and characteristic thicknesses $\eta$ ranging from 4.5 to 27.7 nm.

Traditionally, the peak shift in the particle size distribution of minerals is attributed to Ostwald ripening. One problem with the existing theory is that a size distribution generated from Ostwald ripening should be highly skewed towards larger sizes (e.g.^41^), which apparently contradicts actual measurements (Fig. 2) showing that the peak is skewed towards smaller sizes. In addition, Ostwald ripening is an irreversible process in which larger crystals grow at the expense of smaller ones, ultimately leading to a sharply peaked distribution around a single particle size. To our knowledge, however, a sharply peaked distribution has never been observed for clay particles. In contrary, data show that the size distribution broadens with increasing metamorphic grade of clay samples (Fig. 2). In addition, it is often assumed that the Ostwald ripening of clay could take place over a geological time of millions of years^5^. It is difficult to imagine that a clay-water reaction would not reach equilibrium over such a long time scale, given the fact that clays have relatively fast reaction rates due to their high reactive surface areas and are usually modeled as secondary mineral phases in equilibrium with a contacted geofluid (e.g.^39^). All these arguments point to a possibility that Ostwald ripening may not be a relevant underlying mechanism for describing the particle size distribution of clays. Interestingly, our model provides a reasonably consistent explanation for all of the observed features. As shown in Fig. 2, the skew of the particle size distributions towards smaller sizes is a natural outcome of a random fragmentation process, which we inferred from our analysis of fluctuations in Section “Layer stacking and the Ising model”. In contrast with Ostwald ripening, our model implies that a clay aggregate with a broad particle size distribution can be a thermodynamically stable ensemble which can be preserved over a geological time scale as long as the environment for mineral formation remains relatively unchanged. As prograde metamorphosis progresses, we expect a progressive peak shift and a peak broadening of the clay particle size distribution (Fig. 2) because an elevated temperature and pressure should strengthen clay interlayer interactions (Section “Correlation between layer extension and clay composition”).

In summary, the commonly observed small clay particles can be related to the lack of long-range order in low-dimensional systems. Because of its weak interlayer interacion, a clay mineral can be treated as two separate low-dimensional systems: a 2D system for the individual layers and a quasi-1D system for the layer stacking. The layer stacking in a mixed-layer clay can be described by a 1D Ising model while the limited 2D extension of an individual phyllosilicate layer can be described by an XY-like model. This simple yet powerful treatment allows for a systematic prediction and explanation of the limited dimension of clay particles, the origin of the particle size distribution, the compositional variation of an interstratified clay, and the transition from small illite crystallites to large muscovite crystals. Clay minerals thus provide a useful model system for studying transitions between 1D, 2D, and 3D systems in crystal growth.

## References

[1] Meunie A (2006). Why are clay minerals small?. Clay Miner..

[2] Murray HH (2006). Applied Clay Mineralogy: Occurrences, Processing and Applications of Kaolins, Bentonites, Palygorskite, Sepiolite, and Common Clays.

[3] Warr LN, Nieto F (1998). Crystallite thickness and defect density of phyllosilicates in low temperature metamorphic pelites: A TEM and XRD study of clay mineral crystallinity-index standards. Can. Mineral..

[4] Altaner SP, Ylagan RE (1997). Comparison of structural models of mixed-layer illite/smectite and reaction mechanisms of smectite illitization. Clays Clay Miner..

[5] Eberl DD, Srodon J, Kralik M, Taylor BE, Peterman ZE (1990). Ostwald ripening of clays and metamorphic minerals. Science.

[6] Drever J. I (1982). The Geochemistry of Natural Waters.

[7] Wang Y, Xu H (2006). Geochemical chaos: Periodic and nonperiodic growth of mixed-layer phyllosilicates. Geochim. Cosmochim. Acta.

[8] Zen E (1967). Mixed-layer minerals as one-dimensional crystals. Am. Mineral..

[9] Brigatti MF, Mottana A (2011). Layered Mineral Structures and their Application in Advanced Technologies.

[10] Giese RF (1978). The electrostatic interlayer forces of layer structure materials. Clays Clay Miner..

[11] Honorio T, Brochard L, Vandamme M, Lebée A (2018). Flexibility of nanolayers and stacks: Implications in the nanostructuration of lays. Soft Matter.

[12] Pradhan SM, Katti KS, Katti DR (2014). Evolution of molecular interactions in the interlayer of Na-montmorillonite swelling clay with increasing hydration. Int. J. Geomech..

[13] Zhu TT, Zhou CH, Kabwe FB, Wu QQ, Li CS, Zhang JR (2019). Exfoliation of montmorillonite and related properties of clay/polymer nanocomposites. Appl. Clay Sci..

[14] Hohenberg PC (1967). Existence of long-range order in one and two dimensions. Phys Rev..

[15] Mermin ND, Wagner H (1966). Absences of ferromagnetism or antiferromagnetism in one- or two-dimensional isotropic Heisenberg models. Phys. Rev. Lett..

[16] Landau LD, Lifshitz EM (1980). Statistical Physics Part I.

[17] Peierls R, Born M (1936). On Ising’s model of ferromagnetism. Math. Proc. Camb. Philos. Soc..

[18] van Hove L (1950). Sur L’intégrale de Configuration Pour Les Systèmes De Particules À Une Dimension. Physica.

[19] Chaikin PM, Lubensky TC (2013). Principles of Condensed Matter Physics.

[20] Halperin BI (2019). On the Hohenberg–Mermin–Wagner theorem and its limitations. J. Stat. Phys..

[21] Hong S. S, Yu J. H., Lu D, Marshall A. F., Hikita Y, Cui Y, Hwang H. Y (2017). Two-dimensional limit of crystalline order in perovskite membrane films. Sci. Adv..

[22] Kosterlitz JM, Thouless DJ (1973). Ordering, metastability and phase transitions in two-dimensional systems. J. Phys. C Solid State Phys..

[23] Katti DR, Matar MI, Katti KS, Amarasinghe PM (2009). Multiscale modeling of swelling clays: A computational and experimental approach. KSCE J. Civ. Eng..

[24] Ising E (1925). Beitrag zur Theorie des Ferromagnetismus. Z. Physik.

[25] Baxter RJ (1982). Exactly Solved Models in Statistical Mechanics.

[26] Yilmaz M. B, Zimmermann F. M (2005). Exact cluster size distribution in the one-dimensional Ising model. Phys. Rev. E.

[27] Tenchov B, Yanev T (1986). Weibull distribution of particle sizes obtained by uniform random fragmentation. J. Colloid Interface Sci..

[28] Aja SU, Rosenberg PE (1992). The thermodynamic status of compositionally-variable clay minerals: A discussion. Clays Clay Miner..

[29] Aagaard P, Helgeson HC (1983). Activity/composition relations among silicates and aqueous solutions: II. Chemical and thermodynamic consequences of ideal mixing of atoms on homological sites in montmorillonites, illites, and mixed-layer clays. Clays Clay Miner..

[30] Blanc P, Bieber A, Fritz B, Duplay J (1997). A short range interaction model applied to illite/smectite mixed-layer minerals. Phys. Chem. Miner..

[31] Blanc P, Vieillard P, Gailhanou H, Gaboreau S, Caucher E, Fialips CI, Made B, Giffaut E (2015). A generalized model for predicting the thermodynamic properties of clay minerals. Am. J. Sci..

[32] Gailhanou H, Blanc P, Rogez J, Mikaelian G, Kawaji H, Olives J, Montouillout V, Grenèche J-M, Vieillard P, Gaucher EC, Fialips CI, Madé B (2019). Thermodynamic properties of mixed-layer illite-smectite by calorimetric methods: Acquisition of the enthalpies of mixing of illite and smectite layers. J. Chem. Thermodyn..

[33] Lippmann F (1977). The solubility products of complex minerals, mixed crystals, and three-layer clay minerals. N. Jb. Miner. Abh..

[34] Sakuma H, Suehara S (2015). Interlayer bonding energy of layered minerals: Implication for the relationship with friction coefficient. J. Geophys. Res. Solid Earth.

[35] Christidis GE, Huff WD (2009). Geological aspects and genesis of bentonites. Elements.

[36] Illing B, Fritschi S, Kaiser H, Klix CL, Maret G, Keim P (2017). Mermin–Wagner fluctuations in 2D amorphous solids. PNAS.

[37] Sposito G, Skipper NT, Sutton R, Park S-H, Soper AK, Greathouse JA (1999). Surface geochemistry of the clay minerals. PNAS.

[38] Vidal O, Dubacq B (2009). Thermodynamic modelling of clay dehydration, stability and compositional evolution with temperature, pressure and H_2_O activity. Geochim. Cosmochim. Acta.

[39] Helgeson HC, Garrels RM, Mackenzie FT (1960). Evaluation of irreversible reactions in geochemical processes involving minerals and aqueous solutions-II. Applications. Geochim. Cosmochim. Acta.

[40] Nakazawa H, Yamada H, Fujita T (1992). Crystal synthesis of smectite applying very high pressure and temperature. Appl. Clay Sci..

[41] Vengrenovitch RD (1982). On the Ostwald ripening theory. Acta Metall..

